# Associations Between Academic Stress, Mental Distress, Academic Self-Disclosure to Parents and School Engagement in Hong Kong

**DOI:** 10.3389/fpsyt.2022.911530

**Published:** 2022-07-14

**Authors:** Esther Pui Yung Chyu, Ji-Kang Chen

**Affiliations:** Department of Social Work, The Chinese University of Hong Kong, Hong Kong, Hong Kong SAR, China

**Keywords:** academic stress, mental distress, academic self-disclosure to parents, school engagement, Hong Kong

## Abstract

Numerous studies have indicated that academic stress is associated with various detrimental personal physical and emotional outcomes; however, relatively few studies have explored how academic stress affects adolescents' interactions with their significant others in families and schools, which are two important social systems for school-age adolescents. In addition, there are also few studies examining how academic stress influences adolescents' self-disclosure to parents and school engagement in East Asian districts particularly in Hong Kong, where the level of academic stress among adolescents is high. This study examines how academic stress affects mental distress, academic self-disclosure to parents and school engagement and explores gender differences in the risk for the outcomes of academic stress. One thousand and eight hundred and four students from eight secondary schools in Hong Kong participated in this study. The results indicate that academic stress has a significant association with all three outcomes, but the correlation with school engagement is positive, which is contrary to the findings of most previous studies. The possible reasons for such positive association are discussed. In addition, the model can be applied to both genders, but females are more susceptible to the detrimental outcomes of academic stress by suffering a higher level of mental distress. This study suggests that academic stress should be an important entry point to tackle adolescents' mental distress while interventions should be targeted at females who are experiencing a higher level of mental distress. In addition, in view of the significant associations between academic stress and self-disclosure to parents, as well as between academic stress and school engagement, suggestions are provided to families and schools on how to proactively provide support to those students who are experiencing academic stress.

## Introduction

Academic stress is a growing and alarming threat to young people around the world (1). Research has indicated that adolescents across age groups, genders and cultural contexts are increasingly affected by academic stress (2–4). Empirical findings have also suggested that tremendous academic stress has detrimental impacts on mental wellbeing in young people, including somatic syndromes (5–7), anxiety (3, 8, 9), depression (3, 8, 9), suicidal attempts (10, 11), and addictive habits (12, 13). However, the sample of most of these studies is college students, while high-school students are seldom included in previous studies to examine how academic stress affects their mental health. Although mental health issues among high-school students are well-documented in Hong Kong (14, 15), we are not sure whether these mental health problems are under the influence of academic stress. With this concern, the current study will examine how academic stress affects the level of mental distress among high-school students. The empirical findings of the present study can not only inform youth counselors about the effective way and entry point for handling mental distress among school-age students but also inform policy makers about the necessity of combating academic stress, as tremendous academic stress may have a great effect on students' mental wellbeing.

In addition, spillover theory and the ecology perspective inform us that emotional experiences in one setting will affect what happens in the other settings (16–18). Academic stress, one of the prominent sources of stress among school-age students, will likely affect adolescents' behavior in family and school settings, which are two primary contexts for personal development. However, there is a lack of findings showing how academic stress impacts school-age adolescents' behavior in school and family. In addition, theories and empirical studies have shown that there is a contrasting relationship between academic stress and school engagement in the Western context (19, 20), little is known how academic stress impacts one's involvement in school in East Asian societies, where the school context is quite different from Western societies in terms of school curriculum, teacher-student ratio, and class size. In sum, the primary goal of the current study is to provide observed evidence on the relationship between academic stress and high school students' mental health, interaction with parents and school engagement. In addition, the effect of gender is also examined to determine which gender is at a greater risk of the effects of academic stress.

## Literature Review

### Academic Stress and Mental Distress

Research findings have indicated that academic stress is strongly associated with poor academic performance and procrastination (21, 22), physical illness (5–7), symptoms of mental distress (3, 8, 9, 23–25), suicidal ideations and attempts (10, 11), and addictive behaviors (12, 13). Among these detrimental outcomes, mental distress has received a great deal of attention from researchers and youth counselors. According to the World Health Organization, an estimated 20% of adolescents worldwide experience mental health problems (26). Empirical studies have indicated that academic stress is strongly associated with mental health issues, irrespective of the geographic locations or courses of study (3, 27, 28). However, the samples in most of these studies were college students or undergraduates who were studying a specific subject, such as medicine, or in a specific form, such as college freshmen (8, 29, 30). In Hong Kong, a few studies have examined how academic stress triggers anxiety in elementary students (31, 32). Surprisingly, adolescents in high schools are underresearched in previous studies, particularly in Hong Kong. In addition, relatively few studies have investigated how academic stress among adolescents in high schools impacts their mental wellbeing. Adolescence, described as a period of “storm and stress” (33), is a period during which individuals are particularly vulnerable to academic stress (27). Their identity and values are somehow defined by their academic achievement, particularly through the evaluation and comments from their significant others, such as parents and teachers (34, 35). In Chinese culture, families tend to place a strong emphasis on academic excellence and regard academic achievement as one of the few avenues for upward mobility and bring honor to one's family (36, 37). In addition, children are socialized to be hypersensitive to the judgment of others, especially superiors such as parents and teachers. Therefore, in the context of academic stress, Asian students tend to put pressure on themselves to excel academically, and they also strive hard to meet the academic expectations of significant others, such as parents and teachers (38, 39). Hence, when compared to their counterparts in the West, students in Hong Kong are more susceptible to academic stress triggered by high expectation from family and teachers or from themselves who are striving to satisfy their parents' educational aspiration (38, 39). On the other hand, unlike college students who have almost secured a university degree, high school students in Hong Kong are facing an exit certificate examination; they may consider this period a critical juncture or fateful moment in their life, and it is of great importance to define their future (27). Consequently, adolescents in high schools have more opportunities to be exposed to academic stress and are more susceptible to its detrimental effects on their mental health. However, few studies have provided empirical evidence of how academic stress impacts mental distress in adolescents. Based on previous research, this study hypothesizes that students experiencing academic stress are more likely to develop mental distress.

### Academic Stress and Its Spillover Effects to Other Social Systems

The ecological perspective advises that the social systems of adolescents are interconnected (18). The emotional experiences of high school students may inevitably affect and shape their behaviors in other social settings. The process by which experience in one setting influence behavior or experiences in other contexts is often referred to as spillover (16). The concept of spillover has been widely adopted to explain the linkages between work stress and family experience. For example, a man who is experiencing overwhelming work-related stress may exhaust his energy dealing with that stress and be unable to focus or pay attention, which may adversely affect the frequency and quality of his interactions with his family members (16, 17). Although the concept of spillover offers a useful framework to examine the association between work stress and family experiences, it is rarely applied to examine how academic stress impacts the behaviors of high school students in their other social settings. To the best of our knowledge, the current study is one of the first to examine how academic stress affects interaction and engagement in family and school, which are the two primary contexts for their personal development. The findings of this study can not only provide empirical evidence of whether spillover theory can be applied in academic stress but also enrich theoretical knowledge about the effects of academic stress.

#### Academic Self-Disclosure to Parents

Academic self-disclosure to parents refers to the verbal communication of their thoughts, feelings and experiences in academic areas by adolescents to their parents (40, 41). Self-disclosure, denoting one's willingness and actual behavior in communication, is an imperative basis for family interaction (42). In addition, adolescents' academic self-disclosure to parents plays an important role in allowing parents to have the information they need in a timely manner, enabling them to provide support and guidance to their children who are experiencing academic stress (42). In contrast, low level self-disclosure with parents may impede parents from knowing the academic difficulties that their children are facing (40). A question is what leads adolescents to not disclose their academic difficulties to their parents. Objective self-awareness theory explains that a negative mood caused by a discrepancy between the perceived standard and individual performance may foster withdrawal and inhibit disclosure (41). In addition, uncertainty is regarded as an important emotion governing self-disclosure (43). When adolescents feel vulnerable and anxious about the possible response on the part of the listener, the chance of self-disclosure is reduced (43). In other words, academic stress, a negative emotional state of adolescents, may induce hesitation and worry, which may in turn inhibit self-disclosure. In addition, when overwhelming academic stress accumulates to the point of burnout, students may become withdrawn and passive to disclose their academic issues to their significant others, including their parents (2, 44). Although the above theory suggests that academic stress may influence the level of academic self-disclosure to parents, to the best of our knowledge, no study has provided empirical evidence showing whether academic stress affects self-disclosure to parents. Based on the literature, this study hypothesizes that academic stress is negatively associated with adolescents' academic self-disclosure to parents.

#### School Engagement

School engagement describes students' participation and involvement in the school setting. Although there are different definitions, researchers generally agree that school engagement is a multidimensional construct that is usually composed of three components (45), (1) cognitive engagement, which refers to students' investment in schoolwork, as well as their thoughtfulness, willingness to learn and willingness to make the necessary effort while studying; (2) emotional engagement, which refers to students' enjoyment of and interest in school-related challenges and their emotional reactions to their teachers and classmates; and (3) behavioral engagement, which refers to students' presence at school and compliance with school discipline rules (45). Studies on school engagement have consistently shown that engagement in high school brings favorable outcomes, including better academic achievement, positive teacher-student relationships, healthy psychological wellbeing, and even long-term positive benefits, such as better job opportunities and greater life satisfaction (46). In contrast, low engagement in school is associated with school dropout, a lack of motivation to learn and psychological distress (47).

Self-determination theory argues that the satisfaction of three basic psychological needs, i.e., competency, autonomy and relatedness, is essential for maintaining intrinsic motivation, and such motivation can foster participation and engagement in social systems (48, 49). According to this theory and empirical findings, when students perceive a high level of stress in academics, they may experience a sense of failure in meeting academic demands. This may lead to feelings of incompetency, lack of confidence in their own ability to achieve academic success, and inability to connect with peers due to their own psychological distress. Thus, the three basic psychological needs are not satisfied. As a result, intrinsic motivation is inhibited, which may foster disengagement from school (19, 20, 49). The negative association between stress and school engagement was supported by several empirical studies conducted in the Western context (20). Nevertheless, the literature on stress posits that stress can have facilitative effects by motivating the individual to work hard to perform well in stressful situations if the stress level is still within the range in which coping remains possible (19). In other words, stress can have an activating effect, not only an inhibitory influence, on one's behavior and performance. A study has indicated that the more students report activating test anxiety, the greater they are engaged in school by paying more attention and participating more in lessons. In sum, these different empirical studies have contrasting predictions about how academic stress influences one's engagement in school. Most of these studies are conducted in Western societies, where the school system and context may vary substantially from those in East Asian countries with regard to the curriculum, class size and teacher-student ratio (46). Little is known about how academic stress affects school engagement in an East Asian context. Based on the literature, this study hypothesizes that academic stress is negatively associated with school engagement.

### Effect of Gender

According to the literature on stress, gender plays a crucial role in predicting stress and stress escalation. Although most studies have indicated that girls experience a higher level of academic stress (50, 51) and suffer more from the psychopathology associated with academic stress, the results of some studies have suggested that gender does not play a significant role in predicting stress and is not associated with academic stress (1, 52). In addition, some studies have indicated that girls used to have a higher level of school engagement than boys (53), particularly as reflected in behaviors such as punctuality and regularly doing homework, which have traditionally been considered female characteristics (54). However, findings from one study suggested that test anxiety and stress have positive associations with school disengagement in both boys and girls (20). To extend our knowledge of how gender influences the connections between academic stress and its outcomes, a gender comparison was conducted in this study. Such an examination can not only advance our theoretical knowledge but also provide evidence that can be used to identify high-risk groups, facilitating the implementation of targeted prevention programs.

## Current Study

### Participants

The data used in this study were collected from secondary 4–6 (grades 10–12) students from eight secondary schools in Hong Kong. Convenience sampling strategies were adopted in this study to obtain the data. Although the sample is not strictly representative, the participating schools have covered different bandings, denoting various academic performances, and across different districts in Hong Kong, implying that the students come from different families with different social economic statuses. A total of 2072 secondary 4–6 students were invited to participate in the research, while 258 students or their parents (12.5%) did not give consent to take part. Finally, a total of 1,814 students successfully participated. Ten questionnaires were excluded because they were returned incomplete. As a result, the final data set consisted of 1,804 entries. Of the sample, 789 (43.8%) were boys, 1,012 (56.2%) were girls, and three did not indicate their gender. The grade-level distribution was as follows: 710 (39.4%) students were in secondary 4 (grade 10), 716 (39.7%) students were in secondary 5 (grade 11) and 378 (20.9%) students were in secondary 6 (grade 12).

### Procedure

To assess the adequacy of the scales and ascertain the reliability and validity of the measurement and to test how far the sample can understand the questions, a pilot test was conducted while a total of 124 secondary 4–6 (grades 10–12) students participating. Exploratory factor analysis using the data collected in the pilot test was conducted. The factor loadings of the individual items for each latent variable were generally adequate except for one item from the scale of academic self-disclosure to parents; thus, the item was deleted after the pilot test.

The researchers have conducted a briefing to class-teachers who then helped to deliver a self-administered anonymous survey in the classroom. The students were informed of the background of the study, and they were encouraged to respond truthfully. The questionnaire included 65 items asking about the participants' demographic information and their personal experiences in the school and the family. The survey took approximately 20 min to complete. Written consent was obtained from both students and their parents or guardians before the survey was administered. They were informed that their participation was entirely voluntary, and they were free to withdraw from the study at any time and for any reason. The questionnaire, the related procedures, the informed consent forms and the compliance with ethical practices were reviewed and supervised by the university with which the authors are affiliated.

### Measures

#### Gender

The students were asked to indicate whether they were male or female.

#### Demographic Information

The students were asked to report their demographic background across ten items, including the year of education, age, parents' highest level of educational attainment, occupational background of parents, living arrangements of the family, family financial situation and their academic ranking in the class in the last semester.

#### Academic Stress

In the East Asian context, academic stress is mostly conceptualized as academic expectation stress, in which expectations come from parents, teachers and students themselves (39, 55). In addition, frequent comparisons and excessive demands are the other two dimensions of academic stress (56, 57) that are suggested in the literature. Hence, based on the literature and findings of previous studies, academic stress in this study is first operationalized with five dimensions, namely, academic expectation stress from parents, academic expectation stress from teachers, academic expectation stress from students themselves, frequent comparisons, and excessive demands. Items measuring these five dimensions were from various validated inventories (57–59) and were compiled as the initial scale of academic stress for this study. However, the result of an exploratory factor analysis indicated that there were four indicators of the variable instead of five. The items measuring “frequent comparisons” were finally incorporated into the indicator of “academic expectation stress from students themselves”, while the indicator of “excessive demands” remained even though its factor loading was slightly low.

Based on the findings of the pilot test, seventeen items were used to assess academic stress. These items asked students about their actual feelings regarding different descriptions. The items were rated on a 5-point Likert scale (“1 = totally disagree” to “5 = totally agree”). This latent variable consisted of four subscales with a total of 17 items. The first three subscales were academic expectations stress from parents (five items, factor loading = 0.71), academic expectations stress from teachers (three items, factor loading = 0.76) and academic expectations stress from the students themselves (six items, factor loading = 0.85). The fourth subscale was excessive demands (three items, factor loading = 0.46). All 17 items were selected from the inventories in research studies in Taiwan (47–49). The following are some sample questions: “I blame myself if I cannot meet my parents' academic expectations” (academic expectations stress from parents), “If I have a poor performance in school, I think my teachers are disappointed in me” (academic expectations stress from teachers), “If I cannot meet my own expectations, I am not good enough” (students' academic expectations stress) and “The assessments and examinations are too much for me, and I feel that they are unbearable” (excessive demands). The score for this scale was calculated by summing these 17 items, with a higher score indicating a higher level of academic stress. The Cronbach's alpha coefficient for these items was 0.919.

#### Mental Distress

The items of this latent variable were derived from the Brief Symptom Rating Scale (BSRS). This scale is a Chinese inventory that was developed and validated by Taiwanese psychiatrists and is used as a screening tool to identify common mental health problems (60). The Brief Symptom Rating Scale has 50 items with ten subscales, measuring different mental distress symptoms, namely, somatization, obsession, interpersonal sensitivity, depression, anxiety, hostility, phobia, paranoid, psychoticism and addiction. As depression, anxiety and somatization are common mental distress symptoms among adolescents, these three subscales were then selected to construct a latent variable of mental distress in this study. The item responses were given on a 5-point Likert scale (“1 = never” to “5 = very severe”). The three subscales are (1) depression (seven items, factor loading = 0.91), including the items “I feel lonely” and “I feel hopeless about the future”; (2) anxiety (seven items, factor loading = 0.95), including the items “I am scared” and “I feel unsettled and I cannot sit calmly”; and (3) somatization (five items, factor loading = 0.77), including the items “I feel that it is hard to breathe” and “I have chest pain.” The score for this scale was calculated by summing these 19 items, with a higher score indicating a higher level of mental distress. The Cronbach's alpha coefficient for these items was 0.957.

#### Academic Self-Disclosure to Parents

This variable was measured with five items that asked students about the extent to which they agreed with statements regarding academic self-disclosure to parents. The inventory was translated from the questionnaire developed by Kerr and Stattin (61). These five items were translated from English to Chinese to maintain a consistent language version of the questionnaire, and a standard back-translation procedure was employed to ensure accuracy. The original scale had six items, one of which was deleted after the pilot test because of its low factor loading. The responses to the items were given on a 5-point Likert scale (“1 = totally disagree” to “5 = totally agree”). The results of an exploratory factor analysis suggested that the scale was unidimensional. To build a robust latent structure for academic self-disclosure to parents, these five items were randomly placed into three parcels. The first parcel (factor loading = 0.93) included two items, i.e., “I can discuss my academic issues with my mother/father without feeling restrained or embarrassed” and “My parents try to understand my concerns and views regarding academics.” The second parcel (factor loading = 0.88) also included two items, i.e., “It is easy for me to express my true feelings about academics to my mother/father” and “My parents are good listeners.” The third parcel (factor loading = 0.84) had one item, i.e., “If I experienced academic difficulties, I would tell my mother/father.” The score for this scale was calculated by summing these five items, with a higher score indicating a high level of academic self-disclosure to parents. The Cronbach's alpha coefficient for these items was 0.917.

#### School Engagement

This variable was measured with eight items that asked the students about their views and feelings regarding school engagement. The items were selected from an inventory developed in Taiwan (62). The inventory has 29 items measuring the three dimensions of school engagement, i.e., cognitive, affective and behavioral engagement. The criteria for item selection were based on factor loadings and the content of the items to ensure the scale's reliability and validity. The responses to the items were given on a 5-point Likert scale (“1 = totally not met” to “5 = totally met”). The results of an exploratory factor analysis indicated that there were three factors: (1) cognitive engagement (two items, factor loading = 0.66), including the item “I will try different ways to understand the teacher's lecture during class;” (2) affective engagement (three items, factor loading= 0.66), including the items “I am proud of my school” and “I feel relaxed when I interact with my teachers”; and (3) behavioral engagement (three items, factor loading = 0.54), including the items “I will actively participate in classroom discussions” and “I will take the initiative to ask questions.” The score for this scale was calculated by summing these eight items, with a higher score indicating a higher level of school engagement. The Cronbach's alpha coefficient for these items was 0.831.

#### Plan of Analysis

Descriptive analyses of the variables in this study were first conducted, followed by latent variable structural equation modeling (SEM) with maximum likelihood estimation using the AMOS program (version 27). Confirmatory factor analysis was first conducted to ensure that the measurement model had a good fit (63). Next, the SEM was tested with the full dataset. Cross-group SEM was applied to examine gender differences in the theoretical model. In this comparative analysis, all the factor loadings and the paths of the same model were constrained to be simultaneously equal across genders. Then, the model was tested by releasing the path constraints to determine whether releasing the equality constraint could significantly improve the fit. The model fit was evaluated using SEM incremental fit indices, including the normed fit index (NFI), comparative fit index (CFI), incremental fit index (IFI) and root mean square error of approximation (RMSEA). Typically, an NFI, CFI and IFI above 0.95 and an RMSEA below 0.06 indicate that the model fits the data well (64–66). A number of demographic variables were added to the model as control variables before conducting the SEM analysis. They were family economic status, father's education level and mother's education level.

## Results

### Descriptive Statistics

Table 1 shows the descriptive statistics (means and standard deviations) of the study variables broken down by gender. Table 2 shows the correlations among the four variables. All the variables are positively associated with each other except two, which are both related to academic self-disclosure to parents (academic stress, *r* = −0.090, *p* < 0.01; mental distress, *r* = −0.194, *p* < 0.01). Another negative but non-significant correlation was found between mental distress and school engagement. The strongest association was found between academic stress and mental distress (*r* = 0.459, *p* < 0.01). Two relatively strong associations were found related to school engagement (academic stress, *r* = 0.197, *p* < 0.01; academic self-disclosure to parents *r* = 0.238, *p* < 0.01).

**Table 1:** Descriptive statistics of the research variables.

**Means and standard deviations for each scale (standard deviations in parenthesis)**			
	**Overall**	**Gender**	
		**Male**	**Female**
1. Academic stress^a^	57.76 (11.19)	55.14 (11.62)	59.80 (10.41)
2. Mental distress^b^	40.87 (16.99)	38.10 (15.56)	43.01 (17.74)
3. Academic self-disclosure to parents^a^	14.83 (4.80)	14.59 (4.75)	15.02 (4.84)
4. School engagement^c^	26.37 (4.88)	26.52 (4.93)	26.27 (4.93)

*^a^On a scale: from 1 = “Strongly disagree” to 5 = “Strongly agree”.*

*^b^On a scale: from 1 = “Never” to 5 = “Very severe”.*

*^c^On a scale: from 1 = “Totally not met” to 5 = “Totally met”*.

**Table 2:** Intercorrelations between variables.

	**1**	**2**	**3**	**4**
1. Academic stress	–	0.459**	—.090**	0.197**
2. Mental distress		–	−0.194**	−0.045
3. Academic self-disclosure to parents			–	0.238**
4. School engagement				–

^**^p <0.01.

### Overall Model

The results of the analysis based on the total sample indicated that the model was a good fit for the data [$\chi_{\left(104,\;\text{N}=1,804\right)}^{2}$ = 705.757, *p* < 0.001, with NFI = 0.947, CFI = 0.954, IFI = 0.954, and RMSEA = 0.06].

**Figure 1:**
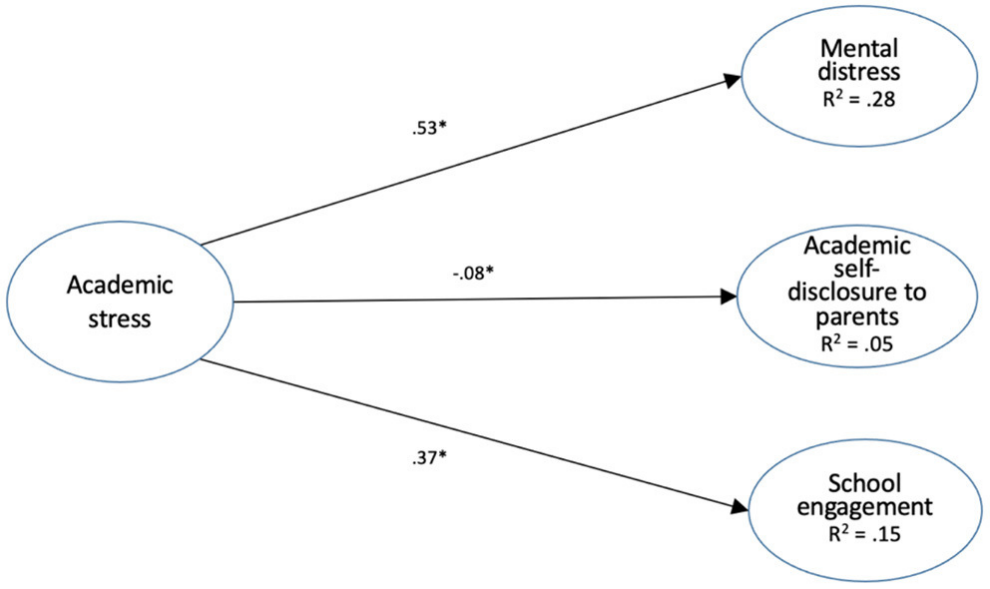
Structural equation modeling of the direct effect of academic stress on mental distress, academic self-disclosure to parents and school engagement (numbers are standardized effects. *Indicates statistical significance at *p* < 0.01.).

Figure 1 shows the paths in the overall model. The three paths to the three endogenous variables were all significant: the path from academic stress to mental distress (β = 0.53, *p* < 0.001), the path from academic stress to academic self-disclosure to parents (β = −0.08, *p* < 0.001), and the path from academic stress to school engagement (β = 0.37, *p* < 0.001).

Overall, the variable of academic stress accounted for 28, 5, and 15% of the explained variances in the dependent variables of mental distress (*R*^2^ = 0.28), academic self-disclosure (*R*^2^ = 0.05) and school engagement (*R*^2^ = 0.15), respectively.

### Gender Comparison

In this analysis, the paths in the same model were constrained to be simultaneously equal in the male and female subgroups. The analysis showed that there was a good fit to the data [χ^2^_(121, N:*males* = 789, *females* = 1, 012)_ = 719.951, *p* < 0.001, with NFI = 0.940, IFI = 0.950, CFI = 0.950, and RMSEA = 0.052].

**Figure 2:**
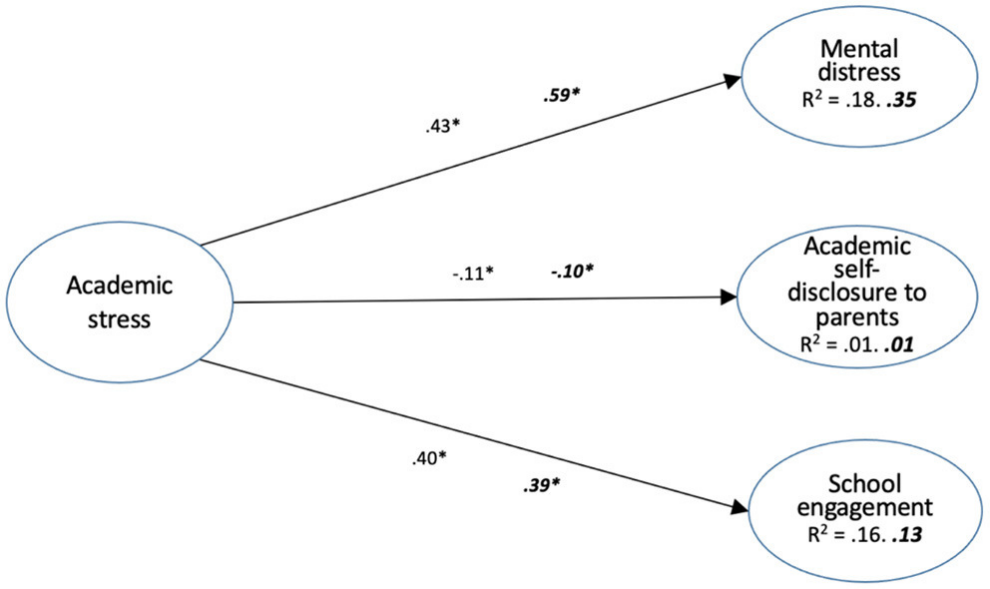
Structural equation modeling of the direct effect of academic stress on males' and ***females'*** mental distress, academic self-disclosure to parents and school engagement (numbers are standardized effects. *Indicates statistical significance at *p* < 0.01).

Next, the model was tested to determine whether releasing the equality constraints on the paths could significantly improve the fit. After releasing path constraints one at a time, it was determined that releasing the constraint between academic stress and mental distress yielded a better fit. The result for the final model with one constraint released was as follows: χ^2^_(120, N:*males* = 789, *females* = 1, 012_) = 705.026, *p* < 0.001, with NFI = 0.942, IFI = 0.951, CFI = 0.951, and RMSEA = 0.052. Figure 2 presents the results of this analysis.

Gender differences emerged in the path between academic stress and mental distress. The association between academic stress and mental distress is stronger among females (β = 0.59) than among males (β = 0.43). Finally, the overall model explained 35% of the variance in mental distress among females (*R*^2^ = 0.35) and 18% of the variance among males (*R*^2^ = 0.18). These results mean that the overall model explains mental distress better for females than for males.

## Discussion

This study examined how academic stress affects mental distress, academic self-disclosure to parents and school engagement among high school adolescents in Hong Kong. A comparison by gender was also performed to determine whether the theoretical model fit both genders.

As hypothesized, the results of the present study indicate that the association between academic stress and mental distress is positive and significant. This finding is consistent with previous empirical studies that suggested that students who experience academic stress are more likely to suffer from various symptoms of mental distress (3, 8, 9, 23). In view of the strong correlation (*r* = 0.46) between academic stress and mental distress in this study, academic stress should indeed be considered a risk factor for mental health problems in adolescents in Hong Kong. In addition, academic stress being measured in this study is referred to as stress triggered by the academic expectations of parents, teachers and adolescent themselves. To satisfy their parents' educational aspiration, teachers' academic demands, and their own scholastic desire, high-school students strive for excellence and success in academic performance, which may push them to the edge of mental distress, especially those examinations that they are facing are crucial to determine their path after high school.

In line with our hypothesis, the results of this study suggest that there is a negative and significant association (*r* = 0.09) between academic stress and academic self-disclosure to parents. Specifically, a higher level of academic stress means that students are unlikely to disclose their academic situation to their parents (40). This finding supports the present theoretical framework and its integration with spillover theory and objective self-awareness theory. When high school students experience academic stress, their distress may spill over to the family system and affect communication with their parents with a decrease in self-disclosure to parents (16). This blockage of communication will prevent parents from being informed of the academic situation and the difficulties experienced by their children in a timely manner (42). These findings also provide empirical data to support the ideas suggested by objective self-awareness theory, that once students are aware of their own situation or performance, they will likely develop a negative mood when they see the discrepancy between their performance and the standard; this, in turn, will inhibit their self-disclosure, especially when they are not sure of their parents' responses (41, 67). Moreover, non-disclosure will mean that parents remain ignorant of the academic situation their child is in, making it difficult for them to provide support and assistance in a timely manner.

In contrast to our hypothesis, the results of this study indicate that academic stress is positively associated with school engagement, i.e., the higher the level of academic stress is, the more adolescents are engaged in school. This result is contrary to most past empirical studies that concluded that there was a negative association between stress and school engagement (19, 20, 46, 68). Although stress, particularly excessive stress, has detrimental impacts on the health and functioning of individuals, it may also have facilitative effects by motivating the individual to work hard or perform well in stressful situations if the stress level is still within the coping range (69, 70). Stress related to examinations or academic issues may motivate students to further engage in school by paying more attention to their learning, putting extra effort into their schoolwork, or increasing their participation in lessons to overcome challenges. In other words, academic stress may be associated with greater school engagement if students have the desire and determination to master their academic challenges.

In addition, school engagement is usually considered a multidimensional construct that has three common components, i.e., cognitive, affective and behavioral engagement (71). However, some school personnel adopt typology to classify students based on their different types of engagement (72), including (1) engagement, (2) strategic compliance, (3) ritual compliance, (4) retreatism and (5) rebellion (72). This typology is based on the nature of and motivation behind school engagement. Based on this classification system and the three different components of school engagement, Conner suggested that there are seven categories, namely, purposeful, full, rational, busy, pleasurable, mental and recreational engagement (73). The findings indicated that most students, even those experiencing stress during high school, display busy engagement, i.e., they consistently work hard and put in effort; however, they rarely enjoy their work (73). This type of engagement is mainly driven by the belief that engagement in school is necessary and beneficial to academic success. In the present study, the positive association between academic stress and school engagement may reflect the fact that the students display this type of school engagement, i.e., busy engagement, in school to address their learning difficulties even if they are experiencing academic stress. This possible explanation of the positive association between academic stress and school engagement takes motivation into consideration, and further investigation should be conducted to substantiate this proposition.

The results of this study show that the theoretical model fit both genders, showing that academic stress predicts mental distress, academic self-disclosure to parents and school engagement. Notably, the findings of this study suggest that girls who are experiencing academic stress are more vulnerable to developing mental distress. This finding is consistent with previous studies that concluded that females are more susceptible to the detrimental effects of academic stress in terms of mental health symptoms (19, 46, 68).

## Implications for Theories, Policies and Practices

Previous studies have suggested that factors from personal, familial and school domains trigger academic stress (2, 52, 74, 75). However, relatively few studies have explored how academic stress in turn affects adolescents' interactions in their school and familial domains. Unlike most previous studies on academic stress that focused mainly on how academic stress affects individual physical and mental health, the present study explored the effects on interpersonal interactions in the family and engagement in the school system, which are two significant systems for adolescents. The findings of the present study support the current theoretical model, particularly when viewed in conjunction with spillover theory, suggesting that academic stress can spill over to other social systems and affect adolescents' interactions with significant others and their participation in the social environment (16, 18). Nevertheless, the directions of the effect are not the same in the family and school systems. Specifically, a higher level of academic stress discourages adolescents from disclosing their academic issues to their parents but promotes their engagement in school. The associations between academic stress and self-disclosure to parents and between academic stress and school engagement may reflect the action of inhibiting and facilitating forces, respectively. Notably, the positive association between academic stress and school engagement is in contrast with the findings of most previous empirical studies (20, 46, 68, 76). These contradictory findings suggest the need for further research into whether the association depends on certain psychosocial mechanisms, such as academic motivation or academic aspiration. Future research might include mediator(s) in the model to elucidate the pathway from academic stress to school engagement. In addition, the dimensions of school engagement, i.e., cognitive, affective and behavioral, could be investigated separately (76), as the antecedents and consequences of the different engagement dimensions may also vary (46).

Although mental health issues among high school students are well-surveyed in Hong Kong, whether academic stress is a possible factor that may influence the level of mental distress has seldom been investigated. This study provides empirical evidence that academic stress can have detrimental effect on individual mental wellbeing among adolescents. In view of the strong correlation between academic stress and mental distress in the present study, academic stress is an important entry point and intervention target to reduce the levels of depression, anxiety and somatization in students. Policymakers should allocate resources to launching campaigns to increase awareness of the harmful effects of academic stress. Education-related social policies should be re-examined by reviewing the curriculum, frequency of examinations and methods of assessment with the aim of reducing the level of academic stress. Different levels of school personnel, including principals, classroom teachers and school counselors, can work together to promote effective coping strategies among their students to reduce academic stress. Such multilevel collaborations can support students' psychological wellbeing in the long term. In addition, social work practitioners can organize psychosocial education on stress management or relaxation exercises for students who experience academic stress, which is a risk factor for developing depression, anxiety and somatization. Finally, although prevention and intervention programs could be provided to both boys and girls, particular attention and dedicated resources should be allocated to girls, who tend to suffer more from the undesirable effects of academic stress in terms of mental health problems.

One of the major findings of the present study is that students who experience a high level of academic stress are less likely to disclose their academic issues to their parents. Parents need to be aware of this association and should remember that they cannot always rely on their children's self-disclosure of their academic difficulties. Hence, school social workers could provide family intervention and sensitivity training to parents and suggest ways in which they can take the initiative to show their concern to their children and detect the academic struggles and stress experienced by their children. Such sensitivity and alertness can facilitate the implementation of early and proactive interventions.

Although school engagement is consistently found to be associated with positive outcomes, such as better academic achievement, positive teacher-student relationships, and higher life satisfaction (46), the positive connection between academic stress and school engagement shown in this study suggests that engagement in school should not be assumed to be an exclusively positive sign. In other words, a high level of school engagement does not imply freedom from or a low level of academic stress. Teachers and school counselors should always determine what is underlying the presence or absence of engagement rather than focusing on engagement behaviors. Is engagement related to academic stress or other factors? With this awareness, individuals who are required to help students will not overlook academic stress as an underlying factor resulting in school engagement.

## Limitations

There are a few limitations of this study that should be considered when interpreting the results. First, this research was based on cross-sectional data; thus, the causal relationships among the variables cannot be ascertained. A longitudinal panel design can be adopted to investigate causality among the variables in this study. Second, our data were based exclusively on adolescents' self-reports. It is possible that the significant associations between the latent variables may be affected by shared method variance if adolescents were the sole respondents. The information collected for academic stress, mental distress, academic self-disclosure to parents and school engagement was self-reported by students, who might have exaggerated their perceptions or underreported their level of academic stress and mental distress due to social desirability bias and the sensitivity of the issue of mental health. Further studies could consider multiple informants, such as parents or teachers, to overcome the issue of shared method variance. Third, although the sample size was large, this study used a convenience sample of students in Hong Kong. Hence, the conclusions should be interpreted with caution and should not be generalized to other age groups or cultural contexts. Fourth, in view of the relatively small correlations between academic stress and academic self-disclosure to parents, the findings should be interpreted with caution. In addition, there is no analysis on how school level, as a cluster, influences the variables that were examined. Therefore, how the school may affect the association between academic stress and its associated effects, particular school engagement, is not fully understood. Finally, the data were collected during the COVID-19 pandemic, and students may encounter insecurity about schooling and public assessment, which may have affected their experiences in school and their level of academic stress. Hence, the results of this study should be interpreted with caution.

## Conclusion

In summary, the present study provides empirical evidence of the effects of academic stress on mental distress, academic self-disclosure to parents and school engagement in Hong Kong. The findings indicate that academic stress has significant associations with all these effects. In addition, the results also reflect that the pattern of the association and effects on these three endogenous variables are similar across genders, although females who experience academic stress tend to develop more mental distress. This implies that policies and social work intervention can be applied to both genders, but more attention should be given to females experiencing academic stress.

## Data Availability Statement

The raw data supporting the conclusions of this article will be made available by the authors, without undue reservation.

## Ethics Statement

The studies involving human participants were reviewed and approved by the Chinese University of Hong Kong Survey and Behavioral Research Ethics. Written informed consent to participate in this study was provided by the participants' legal guardian/next of kin.

## Author Contributions

EC and J-KC: conceptualization, methodology, and data analysis. EC: writing and project administration. J-KC: review—editing. All authors contributed to the article and approved the submitted version.

## Conflict of Interest

The authors declare that the research was conducted in the absence of any commercial or financial relationships that could be construed as a potential conflict of interest.

## Publisher's Note

All claims expressed in this article are solely those of the authors and do not necessarily represent those of their affiliated organizations, or those of the publisher, the editors and the reviewers. Any product that may be evaluated in this article, or claim that may be made by its manufacturer, is not guaranteed or endorsed by the publisher.
